# Single-sEV profiling identifies the TACSTD2 + sEV subpopulation as a factor of tumor susceptibility in the elderly

**DOI:** 10.1186/s12951-024-02456-x

**Published:** 2024-05-03

**Authors:** Nannan Ning, Jianying Lu, Qianpeng Li, Mengmeng Li, Yanling Cai, Hongchun Wang, Jingxin Li

**Affiliations:** 1 Department of Clinical Laboratory, Qilu Hospital of Shandong University, Jinan, China; 2 Shandong Engineering Research Center of Biomarker and Artificial Intelligence Application, Jinan, China; 3 School of Public Health, Shandong University, Jinan, China; 4 Department of Hematology, Weifang People’s Hospital, Weifang, China; 5 Department of Physiology, School of Basic Medical Sciences, Cheeloo College of Medicine, Shandong University, Jinan, China; 6 Guangdong Provincial Key Laboratory of Systems Biology and Synthetic Biology for Urogenital Tumors, Shenzhen Second People’s Hospital, Shenzhen Institute of Translational Medicine), The First Affiliated Hospital of Shenzhen University, Shenzhen, China

**Keywords:** sEV, Aging, TACSTD2, Tumor

## Abstract

**Background:**

Aging is a very complex physiological phenomenon, and sEVs are involved in the regulation of this mechanism. Serum samples from healthy individuals under 30 and over 60 years of age were collected to analyze differences in sEVs proteomics.

**Results:**

Based on PBA analysis, we found that sEVs from the serum of elderly individuals highly express TACSTD2 and identified a subpopulation marked by TACSTD2. Using ELISA, we verified the upregulation of TACSTD2 in serum from elderly human and aged mouse. In addition, we discovered that TACSTD2 was significantly increased in samples from tumor patients and had better diagnostic value than CEA. Specifically, 9 of the 13 tumor groups exhibited elevated TACSTD2, particularly for cervical cancer, colon cancer, esophageal carcinoma, liver cancer and thyroid carcinoma. Moreover, we found that serum sEVs from the elderly (especially those with high TACSTD2 levels) promoted tumor cell (SW480, HuCCT1 and HeLa) proliferation and migration.

**Conclusion:**

TACSTD2 was upregulated in the serum of elderly individuals and patients with tumors, and could serve as a dual biomarker for aging and tumors.

**Supplementary Information:**

The online version contains supplementary material available at 10.1186/s12951-024-02456-x.

## Background

Aging is a dynamic and complex physiological process that involves a decrease in both cellular and systemic functions of the human body and an increase in the occurrence of disease [1–3]. Aging has been identified as a risk factor for cardiovascular diseases, neurodegenerative diseases, tumors and so on [4, 5]. The deeper manifestations of aging include cellular senescence, mitochondrial dysfunction, telomere loss, and genomic instability [6]. Senescent cells do not die immediately, and they release more extracellular vesicles (EVs) [7, 8]. Cellular senescence is the hallmark of aging and involves an alteration of the cellular secretome known as the senescence-associated secretory phenotype (SASP). The SASP results in the secretion of more proteases and proinflammatory cytokines [9–11]. The SASP accelerates aging, induces persistent and low-level inflammation, and increases the body’s susceptibility to disease. In neurodegenerative diseases, the SASP causes astrocyte changes and promotes Alzheimer’s disease [4, 12]. Senescent fibroblasts enhance the growth of epithelial cells and promote the proliferation of breast tumor cells both in vitro and in vivo [7, 13]. Senescent luminal cells highly express tumor-associated calcium signal transducer 2 (TACSTD2) and are associated with prostate cancer [14, 15].

Small extracellular vesicles (sEVs) are EVs 30–100 nm in diameter with characteristic surface proteins such as TSG101, CD81, and CD63 [4, 16, 17]. sEVs contain proteins and various nucleic acids, participate in substance transport and signal communication, and are involved in various physiological and pathological processes [1, 4, 18]. The B7-H3 protein is increased in sEVs released from senescent prostate cancer cells and has become a diagnostic marker for prostate cancer and a new target for immunotherapy [8]. TACSTD2 is upregulated in prostate cancer cells and can be secreted into sEVs to affect receptor cell function [19, 20].

Proximity-dependent barcoding assay (PBA) is a technique for detecting single sEV by profiling individual sEVs via simultaneous detection of hundreds of surface proteins [21]. In this study, we used PBA to analyze serum sEVs from healthy individuals. By comparing samples from youths and seniors, we analyzed differences in protein and sEVs. In addition, we investigated the effect of sEVs on tumor cells with the aim of clarifying the link between aging and tumors at the sEV level.

## Methods and materials

### Human serum samples and mouse serum samples

Human serum samples were collected from people (20–30 years old and 60–84 years old) who came to the hospital for physical examination between March 2021 and June 2022. Serum samples were collected from February to November 2022 from tumor patients newly diagnosed with breast cancer, cervical cancer, colon cancer, esophageal carcinoma, gastric carcinoma, glioma, liver cancer, lung cancer, pancreatic cancer, prostate cancer, rectal cancer, renal carcinoma or thyroid carcinoma. The serum used for the experiments was collected from the remaining serum of the standard biochemical assays.

Blood from mice (C57BL/6, 3–6 months old and 20–24 months old) was collected from the retroorbital plexus. After the blood had clotted at 4 °C, mouse serum was obtained by centrifugation at 2,500 rpm for 15 min.

### sEV extraction and identification

Human serum samples were collected in 50 ml centrifuge tubes and centrifuged at 1,600×g for 20 min at 4 °C, after which the supernatants were transferred to ultracentrifuge tubes (Beckman Coulter, 344,058). After centrifugation at 10,000×g for 20 min at 4 °C, the supernatants were transferred to new ultracentrifuge tubes. Next, the samples were centrifuged at 100,000×g for 60 min at 4 °C (Beckman Coulter, optima XPN100 ultracentrifuge), the supernatants were discarded, and the precipitates were resuspended in PBS. The sEV precipitates were obtained by centrifugation once more under the above conditions. The sEV precipitates were immersed in 2.5% glutaraldehyde fixation solution and imaged with a scanning electron microscope (SEM, Gemini300, Zeiss, Gemany) with the electron beam at a voltage of 3.0 KV and inlens detector. The sEV precipitate was resuspended in PBS buffer and analyzed in nanoparticle tracking analysis (NTA, NanoSight NS300, Malvern Panalytical, Worcester-shire, UK) according to the instruction from the manufacturer.

The sEV precipitates were lysed in RIPA lysis buffer and centrifuged to obtain proteins. The proteins were separated by a 10% or 15% SDS‒PAGE gel and transferred to a PVDF membrane. After the PVDF membrane was incubated with primary antibodies (CD63, Abcam, ab59479; CD81, Abcam, ab109201; TSG101, Abcam, ab125011; Calnexin, Cell Signaling Technology, 2679) and secondary antibody (Beyotime, A0208), the protein bands were visualized in the presence of enhanced chemiluminescence (ECL) reagent.

### Proximity barcoding assay (PBA)

Human serum sEV analysis was performed based on a previously established PBA technique [21, 22]. First, a 96-well reaction plate coated with streptavidin was coated with biotinylated cholera toxin subunit B to capture the phospholipid bilayer of the sEVs. Serum was added to the reaction wells for affinity purification of sEVs, and then PBA probes (oligonucleotide-labelled antibodies) were added and incubated at room temperature for antigen binding. The proteins analyzed were listed in Supplementary Table S1. Next, rolling circle amplification (RCA) products were added to each well of the plate and hybridized with the oligonucleotides in the probes. An extension reaction occurs for oligonucleotides in the probes to obtain a new sequence from the RCA template, which contains the single sEV barcode. The obtained oligonucleotides were used to construct sequencing libraries, and the sequencing steps were performed on the MGI T7 platform to obtain the raw data. Relying on the decoding package of EVisualizer® software, the fastq sequencing data were decoded into single sEV barcodes – protein barcodes – molecular barcodes, which represent the proteomic features of each single sEV. The total protein expression was normalized via the trimmed mean of M-values (TMM) algorithm for subsequent multiple analyses. The flow self-organizing map (FlowSOM) algorithm was utilized to form subpopulations of sEVs, which were then visualized by t-distributed stochastic neighbor embedding (tSNE). For statistical analysis, *Student’s t* test was used for two samples with a normal distribution, and the Mann‒Whitney U test was used for two samples with a in nonnormal distribution.

### TACSTD2 ELISA

The ELISA kit (Shanghai Zcibio technology, ZC-55,258) and serum samples were equilibrated for 30 min at room temperature. Then, 50 μl of serum or standard products was added to a 96-well plate, and 100 μl of TACSTD2 antibody (horseradish peroxidase-labeled) was added to the same well. The reaction plate was sealed and incubated at 37 °C for 60 min. The mixtures were discarded and the reaction plate was washed 5 times with washing solution. Then, 50 μl of substrate A and 50 μl of substrate B were added to the reaction wells and the plate was incubated at 37 °C for 15 min. Then, 50 μl of termination solution was added to the reaction wells, and the plate was placed on a microplate reader to detect the OD values at 450 nm.

### Cell viability

SW480, SW620, HT-29, SW1116, AGS, HepG2, HuCCT1, RBE, Panc1, HeLa, and K562 tumor cells and HUVECs were cultured as standards. The cells were collected and resuspended in serum sEV-free medium. The cells were counted and spread into a 96-well plate with 5,000 cells per well, and then, medium supplemented with or without human serum sEVs (youth serum, senior serum, senior serum with low TACSTD2, or senior serum with high TACSTD2) was added. sEVs extracted from 1 ml of serum were added to one well. The cells were cultured for 48 h, after which CellTiter-Glo reagent (Promega, G7572) was added to each well. After incubation at room temperature for 15 min, the 96-well plate was placed on a microplate reader (lum mode) to determine ATP values.

### Transwell

Tumor cells (SW480, AGS, HuCCT1, and HeLa) were collected, counted and resuspended in serum-free medium. A total of 30,000 cells were seeded in each transwell chamber. The lower chamber contained medium supplemented with 20% sEV-free FBS. sEVs extracted from approximately 3 ml of serum (youth serum, senior serum, senior serum with low TACSTD2, or senior serum with high TACSTD2) were added to one lower room. The cells were cultured for 48 h, washed with PBS buffer and fixed with 4% paraformaldehyde. After washing again, the cells were stained with crystal violet solution and then observed with a microscope.

## Results

### sEV proteomic analysis by PBA

**Fig. 1:**
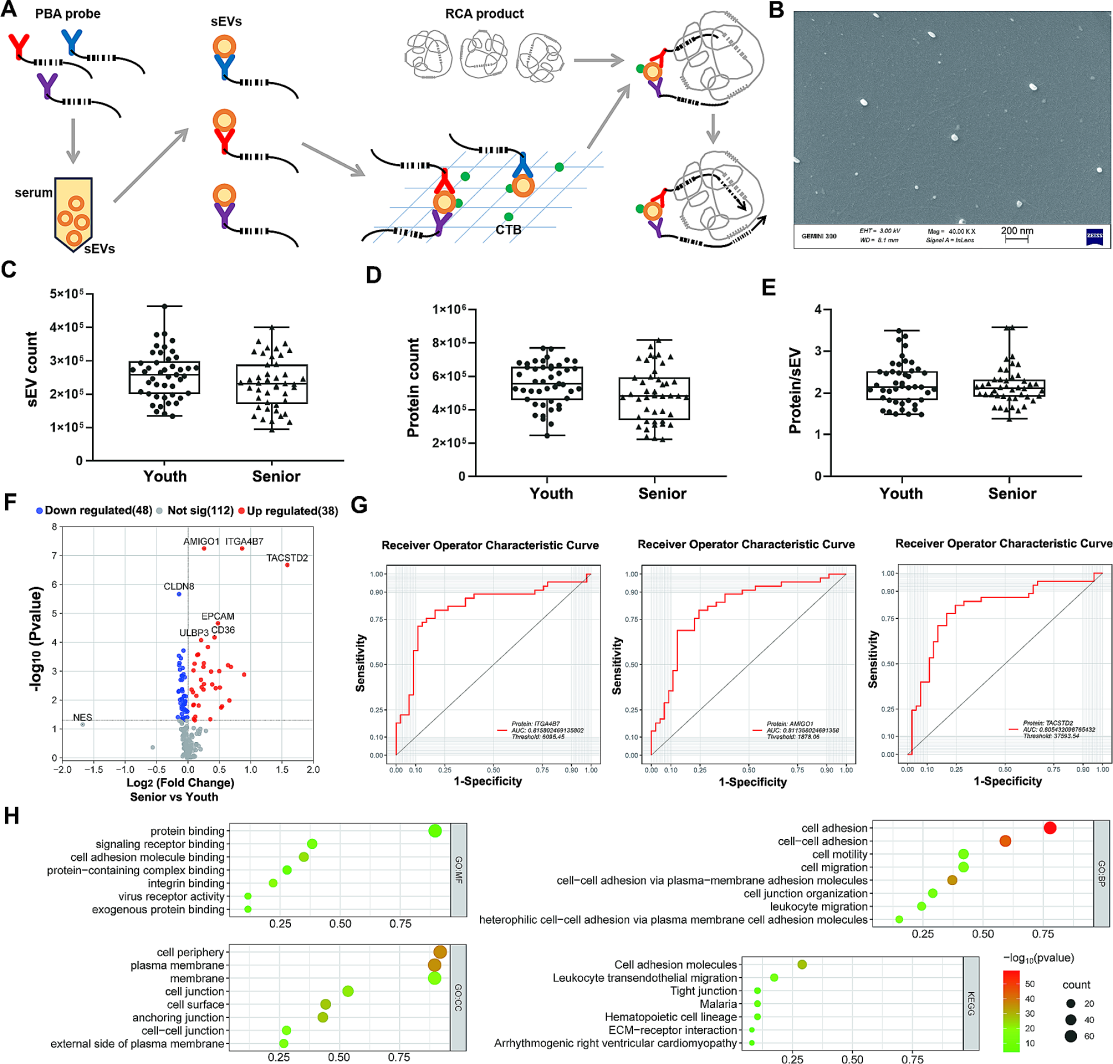
Proteomic data for individual sEVs from youth (20–30 years old, *n* = 44) and senior (60–84 years old, *n* = 45) serum samples were analyzed by PBA. **A**. Schematic diagram of the PBA. PBA probes were antibodies with chemical conjugation of barcoding DNA oligonucleotides, which included 8-nt protein tag to specify each protein and 8-nt molecular tag to distinguish protein molecules. The 3’- part of DNA oligonucleotides on PBA probes were able to hybridize to the RCA product with repeated segments containing the same unique 15-nt EV tag. After extension reaction, the PBA probes on the same EV were incorporated with the EV tag. The whole sequences were employed to analyze the links between EV tag and related protein type and molecular count. Therefore, the protein expression on single EVs was quantified. **B**. Electron microscope image of sEVs captured by PBA. **C**. The number of sEVs detected in each sample. **D**. The number of proteins detected in each sample. **E**. The number of proteins detected per sEV. **F**. Volcano plot of the proteomic data (Senior vs. Youth). **G**. ROC curve of sEV biomarkers for distinguishing between the senior group and the youth group. **H**. Significant GO terms and KEGG pathways for intergroup differential proteins

PBA can be used to analyze sEV surface proteins at single particle resolution via a barcoding method. We tested serum sEVs in healthy youths and seniors and revealed significant differences associated with aging at both the total sEV protein and single sEV feature levels (Fig. 1A and B). In general, in 1 μl of serum from each sample, we detected 4.92 × 10^5^ proteins from 2.31 × 10^5^ sEVs in the senior group and 5.53 × 10^5^ proteins in 2.56 × 10^5^ sEVs for the youth group (Fig. 1C and D). No statistically significant difference was observed. The average number of proteins detected on each sEV in the two groups was 2.23 (youth) and 2.17 (senior) (Fig. 1E). After the calculation of total protein expression data and TMM normalization, proteins with significant differential expression were identified. Compared with those in the youth group, 38 proteins (e.g., ITGA4B7, AMIGO1, TACSTD2, and EPCAM) were upregulated, while 48 proteins (e.g., CLDN8, CLDN6, PLXNB1, and CXCL8) were downregulated in the senior group (Fig. 1F). We plotted ROC curves to evaluate these proteins, and the areas under the curve (AUCs) for ITGA4B7, AMIGO1 and TACSTD2 were 0.8158, 0.8114 and 0.8054, respectively (Fig. 1G). In addition, we uploaded 86 differentially expressed proteins to g: Profiler for GO term and KEGG analyses. We listed the top items, and the results suggested that cell adhesion was the major difference between the two groups (Fig. 1H and Supplementary Table S2).

### sEV subpopulation analysis between youth and senior

**Fig. 2:**
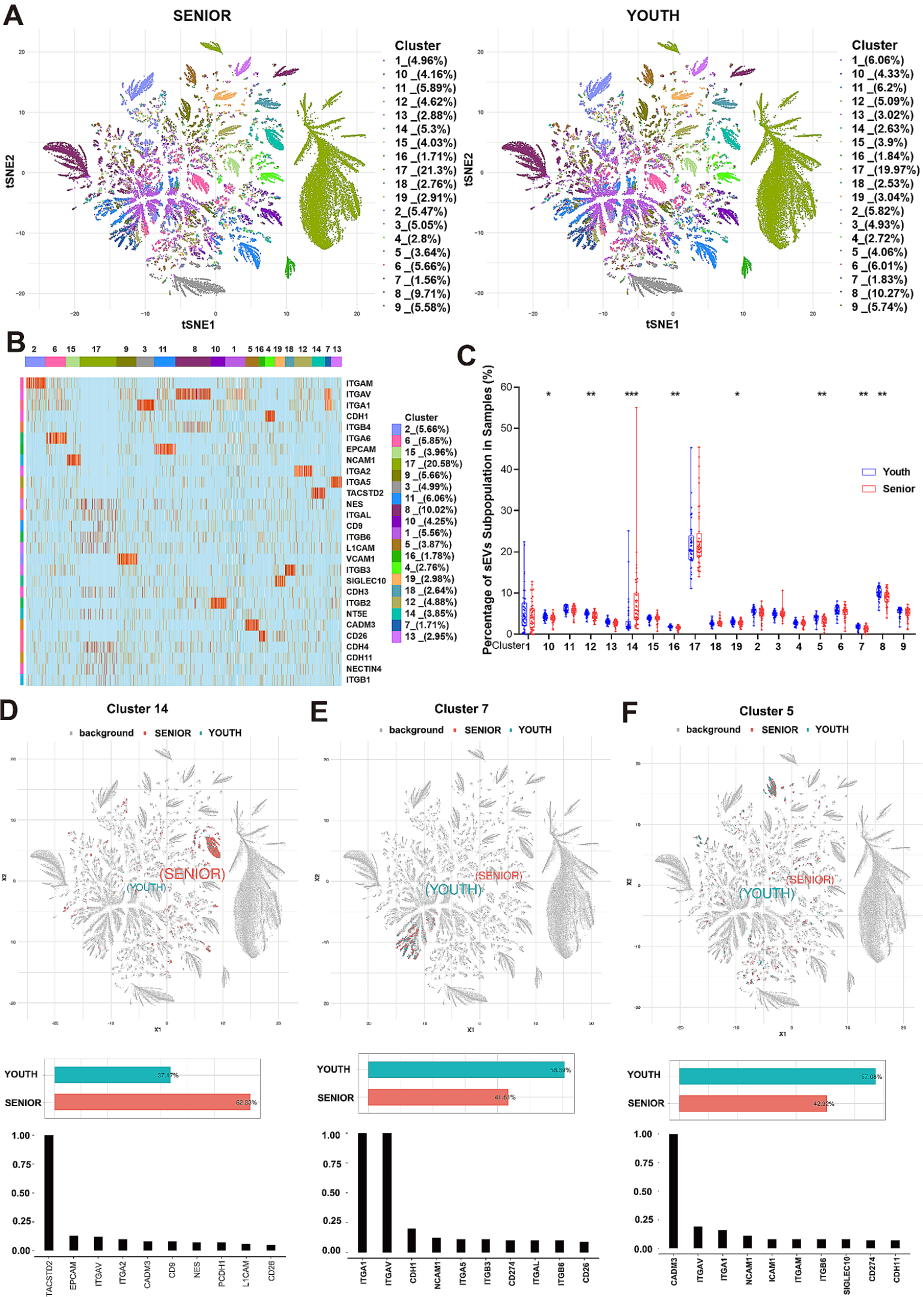
sEV subpopulations in the youth and senior groups. (**A**) The distribution of sEVs in each subpopulation of the two groups. (**B**) The heatmap displays the proteomic markers for each subpopulation. (**C**) The percentage of each subpopulation in the samples. *P < 0.05, **P < 0.01, ***P < 0.001. D-F. Distribution, proportion and protein markers of clusters 14, 7 and 5 in the two groups

To facilitate calculations and analysis, we first filtered and screened the PBA data. We excluded sEVs with fewer than 2 protein markers and randomly selected 1/3 of the sEVs for analysis. These data were downscaled, and the subpopulation were identified by FlowSOM. Then, the clusters were analyzed by tSNE. Based on the similarity of the expressed protein combinations, the sEVs from the two groups of samples were identified as 19 clusters (Fig. 2A, Supplementary Fig. S1 and S2). We quantified the protein expression of each subpopulation and showed the protein abundance of each cluster with heatmap (Fig. 2B). Most clusters had clear marker proteins, and we list these biomarkers in Supplementary Table S3. Cluster 17 had the largest percentage (approximately 20.58%), and the remaining clusters had similar proportions. Among them, clusters 12, 14, 16, 5, 7 and 8 differed significantly between the youth and senior groups (Fig. 2C). The top 3 clusters (14, 7, 5) were shown in Fig. 2D and F. In cluster 14, for which TACSTD2 was the biomarker, the serum TACSTD2 level was significantly greater in the senior group than in the youth group (approximately 1.7 times greater) (Fig. 2D). Clusters 7 and 5 were more abundant in the serum of youth samples and their biomarkers were integrin subunit alpha 1 (ITGA1), integrin subunit alpha V (ITGAV) and cell adhesion molecule 3 (CADM3), respectively (Fig. 2E and F). The three proteins were associated with cell adhesion, which was consistent with previous GO and KEGG analyses. These proteins, especially TACSTD2, might be markers associated with aging.

### Serum TACSTD2 was upregulated in the elderly and in patients with tumors

**Fig. 3:**
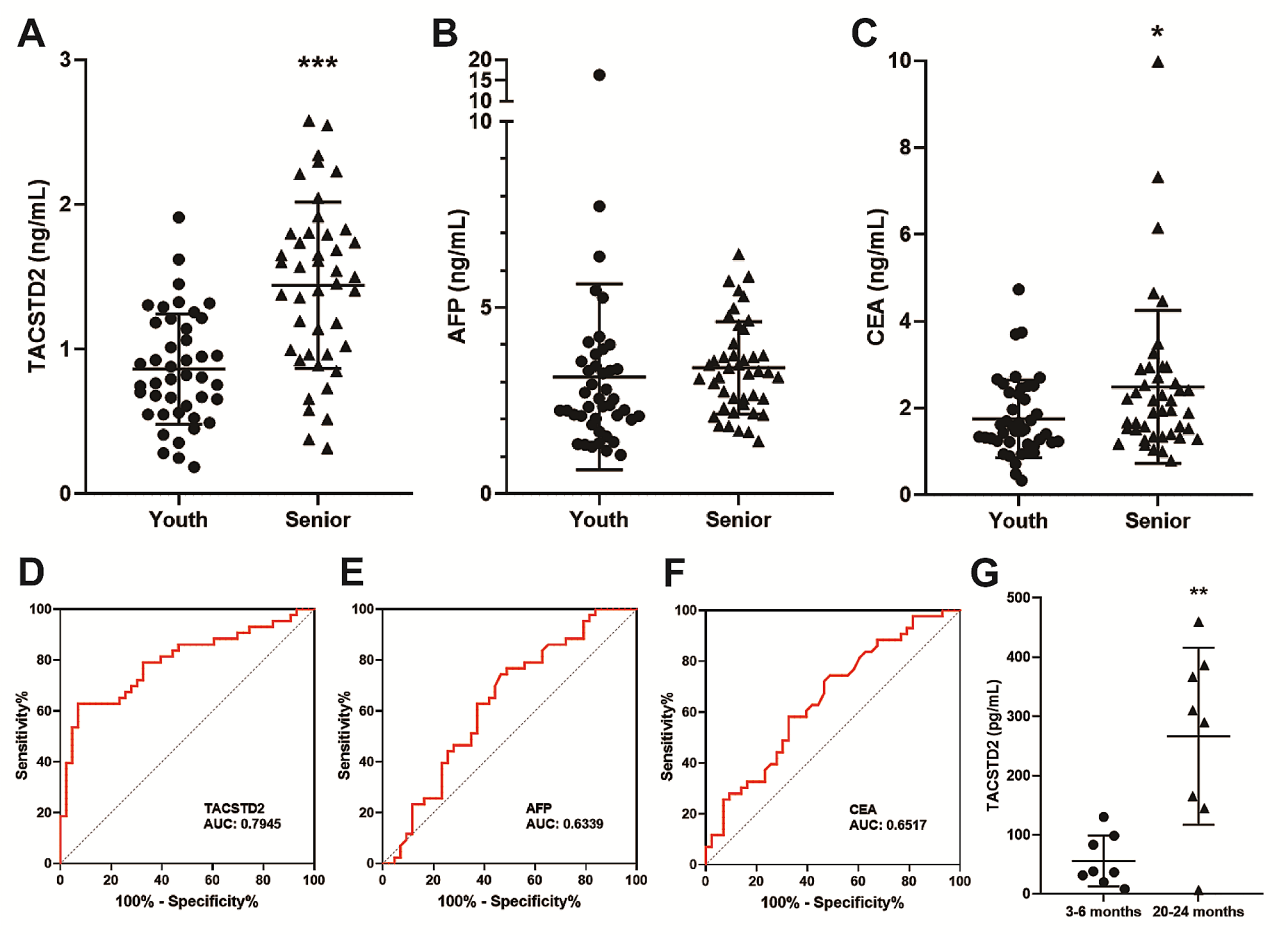
Serum levels of TACSTD2, AFP and CEA in the youth and elderly. **A**-**C**. Serum levels of TACSTD2, AFP and CEA in youth (20–30 years old, *n* = 43) and senior (60–80 years old, *n* = 43) samples. *P < 0.05, ***P < 0.001. **D**-**F**. ROC curves of TACSTD2, AFP and CEA for distinguishing between the senior group and the youth group. **G**. Serum levels of TACSTD2 in youth (3–6 months old, *n* = 8) and senior (20–24 months old, *n* = 8) mice. **P < 0.01

To verify the findings of the sEV analysis, we collected human serum and mouse serum for testing. First, we determined the concentration of TACSTD2 in the serum of healthy individuals using an ELISA kit. The results showed that the serum TACSTD2 concentration in the senior group (1.442 ng/ml) was significantly greater than that in the youth group (0.863 ng/ml) (Fig. 3A). Considering that the elderly are at high risk for tumors, we also measured the levels of the tumor-related markers AFP and CEA in these samples. Compared with the youth group, the senior group contained more CEA, while there was no difference in AFP (Fig. 3B and C). We plotted the ROC curves of the 3 proteins to evaluate their classification value. The AUC values were 0.7945 (TACSTD2), 0.6339 (AFP) and 0.6517 (CEA), which fully suggested that TACSTD2 was a better biomarker (Fig. 3D–F). Moreover, we also collected mouse serum and measured TACSTD2 levels. The average concentration of TACSTD2 in aged mice (266.3 pg/ml) was approximately 4.8 times greater than that in young mice (55.8 pg/ml) (Fig. 3G).

**Fig. 4:**
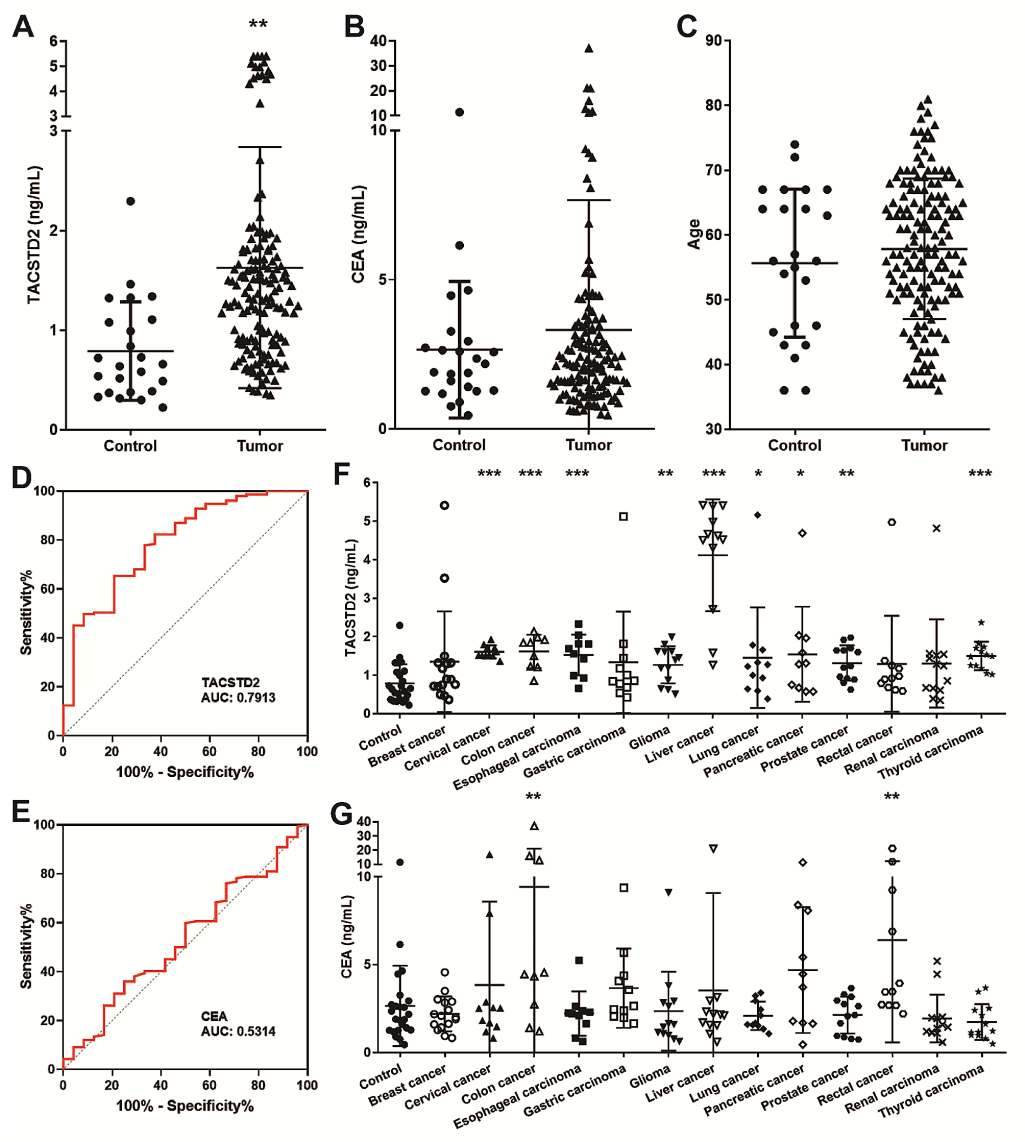
TACSTD2 and CEA in the serum of patients with tumor and healthy controls. **A**-**C**. Serum TACSTD2 and CEA levels and age of tumor patients (*n* = 153) and healthy controls (*n* = 24). **P < 0.01. **D** and **E**. ROC curves of TACSTD2 and CEA in the tumor group from healthy group. **F** and **G**. Serum TACSTD2 and CEA levels in 13 tumor groups and healthy controls (*n* = 24). Breast cancer (*n* = 16), cervical cancer (*n* = 11), colon cancer (*n* = 9), esophageal carcinoma (*n* = 10), gastric carcinoma (*n* = 11), glioma (*n* = 13), liver cancer (*n* = 12), lung cancer (*n* = 11), pancreatic cancer (*n* = 10), prostate cancer (*n* = 13), rectal cancer (*n* = 11), renal carcinoma (*n* = 13), and thyroid carcinoma (*n* = 13). *P < 0.05, **P < 0.01, ***P < 0.001

Previous studies have shown that TACSTD2 is highly expressed in a variety of tumors. Thus, we collected serum from patients with tumors and measured the levels of TACSTD2 and CEA. Serum levels of TACSTD2 were significantly greater in patients (1.628 ng/ml) than in healthy controls (0.789 ng/ml), whereas CEA levels did not differ (Fig. 4A–C). We also generated the ROC curves, and AUC values were 0.7913 (TACSTD2) and 0.5314 (CEA), suggesting that TACSTD2 has good diagnostic value (Fig. 4D and E). In addition, we distinguished the tumor types and performed a comparative analysis. Compared to that in healthy individuals, the serum TACSTD2 level was significantly increased in patients with cervical cancer, colon cancer, esophageal carcinoma, glioma, liver cancer, lung cancer, pancreatic cancer, prostate cancer and thyroid carcinoma, while the traditional tumor marker CEA was only differentially elevated in the serum of patients with colon cancer and rectal cancer (Fig. 4F and G). These findings were consistent with those of previous studies, and TACSTD2 mRNA was highly expressed in various tumors according to UALCAN (Supplementary Fig. S3).

### Serum sEVs in elderly individuals promote tumor cell viability

**Fig. 5:**
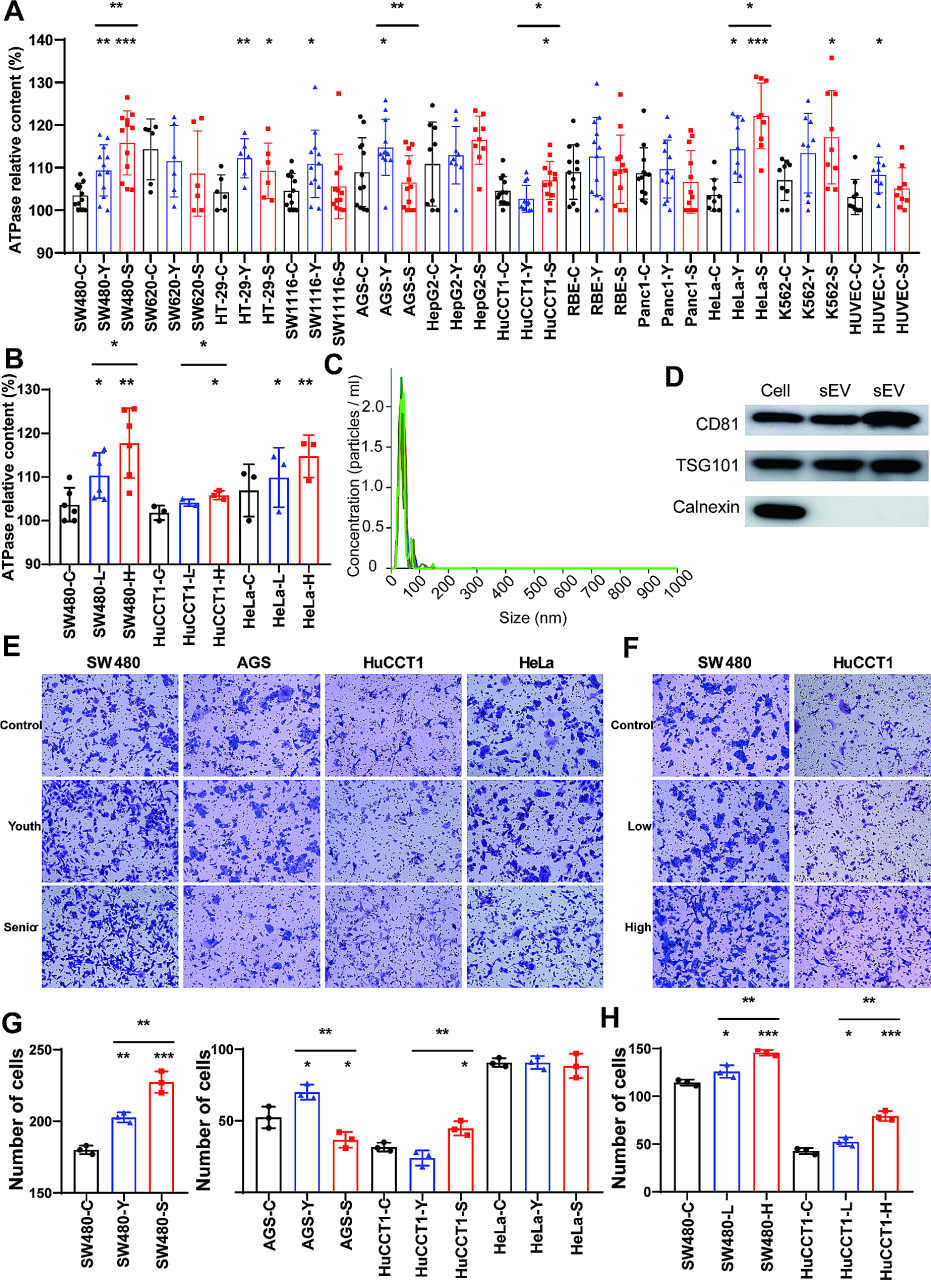
Serum sEVs affect the proliferation and migration of tumor cells. (**A**) Proliferation of tumor cells after treatment with serum sEVs from youth and elderly samples. *P < 0.05, **P < 0.01, ***P < 0.001. (**B**) Proliferation of tumor cells after treatment with sEVs in serum from elderly samples with different TACSTD2 levels. *P < 0.05, **P < 0.01. (**C**) Nanoparticle tracking analysis of serum sEVs. (**D**) Western blot analysis of sEV markers. **E** and **F**. Migration of tumor cells after sEV treatment. **G** and **H**. Statistical graph of migration (including **E** and **F**). *P < 0.05, **P < 0.01, ***P < 0.001

To investigate the effect of serum sEVs on tumor cells, we collected serum from healthy individuals (youths and seniors) and extracted sEVs for cell experiments. We added the extracted sEVs to the cells and found that the sEVs could promoted the proliferation of HUVECs and 6 tumor cell lines (SW480, HT-29, SW1116, AGS, HuCCT1, HeLa, and K562) (Fig. 5A). In SW480, HuCCT1 and HeLa cells, the proliferative effect of sEVs from senior serum was greater than that of sEVs from young individuals. After stratifying senior serum based on TACSTD2 levels, we also found that sEVs from high-level TACSTD2 serum were more able to promote the proliferation of SW480 and HuCCT1 cells (Fig. 5B). We characterized the extracted sEVs before their use in cells. As shown in Fig. 5C and D, sEVs had an average diameter of 40 nm and expressed the specific proteins CD63, CD81 and TSG101. In addition, we performed a transwell experiment to detect cell migration. The results showed that elderly serum sEVs, especially serum with high TACSTD2 levels, could enhance the migration of SW480 and HuCCT1 cells (Fig. 5E–H).

## Discussion

sEVs play important roles in physiological and pathological processes, including aging [23, 24]. For example, sEVs not only affect endothelial cell proliferation and inflammation, but also regulate vascular aging by influencing signaling between endothelial cells and vascular smooth muscle cells [18]. In maternal plasma, miRNAs and proteins in sEVs vary with gestational age [25]. With advances in technology, the identification and analysis of sEVs has expanded to proteomics [26, 27]. sEV proteins can be identified and quantified by flow cytometry after labelling with various fluorescent antibodies [26]. In this study, we identified and quantified hundreds of proteins in serum sEVs using the new technique PBA. We found that several adhesion-associated proteins, particularly TACSTD2, a member of the epithelial cell adhesion molecule (EpCAM) family, were elevated in senior serum sEVs. In addition, we determined the increase in TACSTD2 concentration in senior serum and aged mouse serum. Previously, Crowell’s study suggested that senescent prostate luminal cells highly express TACSTD2 and that the percentage of TACSTD2-positive cells increases with age [14, 28]. By combining different proteins, we analyzed and defined an sEV subpopulation marked by TACSTD2, the expression of which was significantly increased in senior samples.

Aging is an important risk factor for tumorigenesis, and 54% of tumor cases occur in patients over the age of 65 [29]. The characteristics of aging and tumors are similar and even consistent in some respects [30, 31]. According to clinical statistical analysis, aging-related genes are strongly activated in colorectal cancer and are associated with patient prognosis [32]. The removal of senescent macrophages facilitates reduced KRAS-driven tumorigenesis [33]. Interestingly, TACSTD2 is highly expressed in prostate cancer and is associated with the severity and prognosis of the tumor [15, 34]. Moreover, TACSTD2, which is involved in proliferation and invasion, is also upregulated in many tumors and is considered a prognostic marker [35–38]. Here, we measured the serum TACSTD2 levels in 13 types of tumors and found a specific increase in the levels of 9 types of tumors. We also revealed that the diagnostic value of TACSTD2 for tumors was significantly greater than that of CEA, which is consistent with previous lung cancer studies [39]. In addition, the SASP induces cell plasticity and stemness, and sEVs from senescent cells can promote tumor cell proliferation through EphA2 [7, 40]. TACSTD2 not only promotes the proliferation and differentiation of cortical bone-derived stem cells, but also promotes angiogenesis in a paracrine manner [41]. Similarly, we found that senior serum sEVs, especially those from serum with high TACSTD2 levels, promoted tumor cell proliferation and migration.

## Conclusions

In general, PBA is a new high-throughput technique for analyzing sEVs and is ideally suited for primary screening. Relying on the PBA, we analyzed young and senior serum sEVs and identified a TACSTD2 + sEV subpopulation enriched in senior serum. We suggest that elderly serum sEVs can promote the proliferation and migration of tumor cells. TACSTD2 levels are increased in elderly serum and tumor serum and can be used as a biomarker for aging and tumors.

### Electronic supplementary material

Below is the link to the electronic supplementary material.

Supplementary Material 1

## Data Availability

All data generated or analyzed during this study are included in this published article and its supplementary information files.

## Abbreviations

AMIGO1: Adhesion molecule with Ig like domain 1;
CADM3: Cell adhesion molecule 3
CD63: CD63 molecule;
CD81: CD81 molecule;
CLDN6: Claudin 6;
CLDN8: Claudin 8;
CXCL8: C-X-C motif chemokine ligand 8;
EPCAM: Epithelial cell adhesion molecule;
ITGA1: Integrin subunit alpha 1;
ITGA4B7: Integrin subunit alpha 4 beta 7;
ITGAV: Integrin subunit alpha V;
PLXNB1: Plexin B1;
SASP: Senescence-associated secretory phenotype
TACSTD2: Tumor associated calcium signal transducer 2;
TSG101: Tumor susceptibility 101;
