# Supplementary material for: Single-sEV profiling identifies the TACSTD2 + sEV subpopulation as a factor of tumor susceptibility in the elderly
Source: J Nanobiotechnology. 2024 May 3;22:222. doi: 10.1186/s12951-024-02456-x (PMC11067244; doi:10.1186/s12951-024-02456-x)

Supplementary

Table S1. The list of proteins detected (198 proteins).

| ABCG2 | CD36 | CEACAM8 | DSC3 | IL1RAPL1 | ITGB8 | NES | SIGLEC1 |
| --- | --- | --- | --- | --- | --- | --- | --- |
| ADAM10 | CD44 | CLDN1 | DSCAM | IL6 | JAM2 | NFASC | SIGLEC10 |
| ADGRG1 | CD63 | CLDN10 | DSG1 | ILK | JAM3 | NGFR | SIGLEC11 |
| ADIPOQ | CD81 | CLDN11 | DSG2 | ITGA1 | JAML | NLGN1 | SIGLEC14 |
| ALDH1A1 | CD9 | CLDN12 | DSG3 | ITGA11 | KIT | NT5E | SIGLEC5 |
| AMIGO1 | CDCP1 | CLDN17 | DSG4 | ITGA2 | L1CAM | NTRK3 | SIGLEC6 |
| AMIGO2 | CDH1 | CLDN19 | EFNB2 | ITGA2B | LAG3 | PAR2 | SIGLEC7 |
| ANPEP | CDH11 | CLDN3 | EMCN | ITGA3 | LAMP1 | PCDH1 | SIGLEC8 |
| ANXA1 | CDH12 | CLDN4 | EPCAM | ITGA4 | LAMP2 | PCDH15 | SIGLEC9 |
| AXL | CDH13 | CLDN6 | EPHA2 | ITGA4B7 | LGALS9 | PCDH17 | TACSTD2 |
| BCAM | CDH15 | CLDN8 | ERBB2 | ITGA5 | LGR5 | PCDH19 | TENM1 |
| BOC | CDH17 | CLEC1B | ESAM | ITGA6 | LYN | PCDH8 | TENM2 |
| CADM3 | CDH2 | CLEC2A | F11R | ITGA8 | MADCAM1 | PCDHA1 | TENM4 |
| CADM4 | CDH3 | CLEC5A | FN1 | ITGA9 | MCAM | PCDHGC3 | Thy1 |
| CAV1 | CDH4 | CLMP | FOLH1 | ITGAL | MICA | PDCD1 | TICAM2 |
| CCR2 | CDH5 | CLSTN1 | GPA33 | ITGAM | MICB | PDPN | TIM3 |
| CCR6 | CDH6 | CPM | GPC1 | ITGAV | MMP9 | PECAM1 | TIMP2 |
| CD151 | CDHR5 | CSPG4 | GSN | ITGAX | MUC1 | PLAU | TMEM204 |
| CD20 | CDON | CTLA4 | HEPACAM | ITGB1 | MUC16 | PLAUR | TPBG |
| CD22 | CEACAM1 | CXADR | HLA-A | ITGB2 | MUC19 | PLXNB1 | ULBP1 |
| CD24 | CEACAM3 | CXCL16 | HLA-DRA | ITGB3 | MUC4 | PROM1 | ULBP2 |
| CD26 | CEACAM4 | CXCL8 | ICAM1 | ITGB4 | NCAM1 | PTPRJ | ULBP3 |
| CD27 | CEACAM5 | CXCR4 | ICAM2 | ITGB5 | NECTIN1 | RETN | VCAM1 |
| CD274 | CEACAM6 | DSC1 | ICAM3 | ITGB6 | NECTIN3 | SELE |  |
| CD33 | CEACAM7 | DSC2 | ICAM4 | ITGB7 | NECTIN4 | SELL |  |

Table S2. 86 differential proteins

| Protein | Pvalue | Protein | Pvalue | Protein | Pvalue | Protein | Pvalue |
| --- | --- | --- | --- | --- | --- | --- | --- |
| AMIGO1 | 5.69E-08 | MUC4 | 0.000942 | IL1RAPL1 | 0.005093 | CXCR4 | 0.018202 |
| ITGA4B7 | 5.69E-08 | ADAM10 | 0.001002 | MCAM | 0.005368 | CD63 | 0.019919 |
| TACSTD2 | 2.13E-07 | ERBB2 | 0.001033 | EMCN | 0.00713 | ITGA6 | 0.020828 |
| CLDN8 | 2.18E-06 | CEACAM7 | 0.001278 | NLGN1 | 0.00713 | TENM4 | 0.021773 |
| EPCAM | 2.21E-05 | SIGLEC11 | 0.001317 | CD274 | 0.007314 | NTRK3 | 0.022755 |
| CD36 | 6.81E-05 | NGFR | 0.001441 | SIGLEC7 | 0.007501 | ITGB7 | 0.024833 |
| ULBP3 | 8.51E-05 | ICAM1 | 0.001471 | ITGA3 | 0.009019 | DSC1 | 0.025377 |
| CEACAM8 | 0.000147 | CADM3 | 0.001622 | CD22 | 0.009583 | PCDHGC3 | 0.030111 |
| CLDN6 | 0.000194 | ANXA1 | 0.00199 | CLDN1 | 0.009862 | GPC1 | 0.037829 |
| FOLH1 | 0.000265 | ITGB2 | 0.00199 | IL6 | 0.010356 | ICAM3 | 0.039397 |
| ITGA1 | 0.000274 | TENM1 | 0.00199 | MUC19 | 0.010871 | SIGLEC10 | 0.039397 |
| PLXNB1 | 0.000294 | CLDN4 | 0.0028 | CD26 | 0.011408 | EPHA2 | 0.040202 |
| CXCL8 | 0.000359 | CLDN10 | 0.00288 | ABCG2 | 0.012554 | ADGRG1 | 0.04102 |
| PDPN | 0.000527 | JAM2 | 0.003592 | CSPG4 | 0.012855 | AXL | 0.044435 |
| TIMP2 | 0.000533 | CLDN11 | 0.003692 | DSG4 | 0.013801 | ITGB3 | 0.045324 |
| CDH15 | 0.000551 | RETN | 0.003898 | DSC2 | 0.01446 | CDH6 | 0.046229 |
| CLEC2A | 0.000626 | NT5E | 0.004227 | NECTIN3 | 0.01446 | TMEM204 | 0.046229 |
| PCDH1 | 0.000626 | CD81 | 0.004342 | TICAM2 | 0.01515 | ICAM2 | 0.048083 |
| CD9 | 0.000711 | DSCAM | 0.004342 | CLSTN1 | 0.015505 | VCAM1 | 0.049033 |
| DSG1 | 0.000711 | ITGAL | 0.004342 | MMP9 | 0.015505 | PAR2 | 0.049999 |
| ALDH1A1 | 0.000782 | JAML | 0.00483 | ITGA8 | 0.016615 |  |  |
| SELE | 0.000887 | ITGAM | 0.00496 | F11R | 0.017 |  |  |

Table S3. Cluster annotation of serum exosomes

| Subpopulations | Marker1 | Marker2 |
| --- | --- | --- |
| Cluster10 | ITGB2 |  |
| Cluster11 | EPCAM |  |
| Cluster12 | ITGA2 |  |
| Cluster13 | ITGA5 |  |
| Cluster14 | TACSTD2 |  |
| Cluster15 | NCAM1 |  |
| Cluster16 | CD26 |  |
| Cluster18 | ITGB3 |  |
| Cluster2 | ITGAM |  |
| Cluster3 | ITGA1 |  |
| Cluster4 | CDH1 |  |
| Cluster5 | CADM3 |  |
| Cluster6 | ITGA6 |  |
| Cluster7 | ITGA1 | ITGAV |
| Cluster8 | ITGAV |  |
| Cluster9 | UCAM1 |  |

Figure S1. Heat map of AUC values for each protein combination as a biomarker.


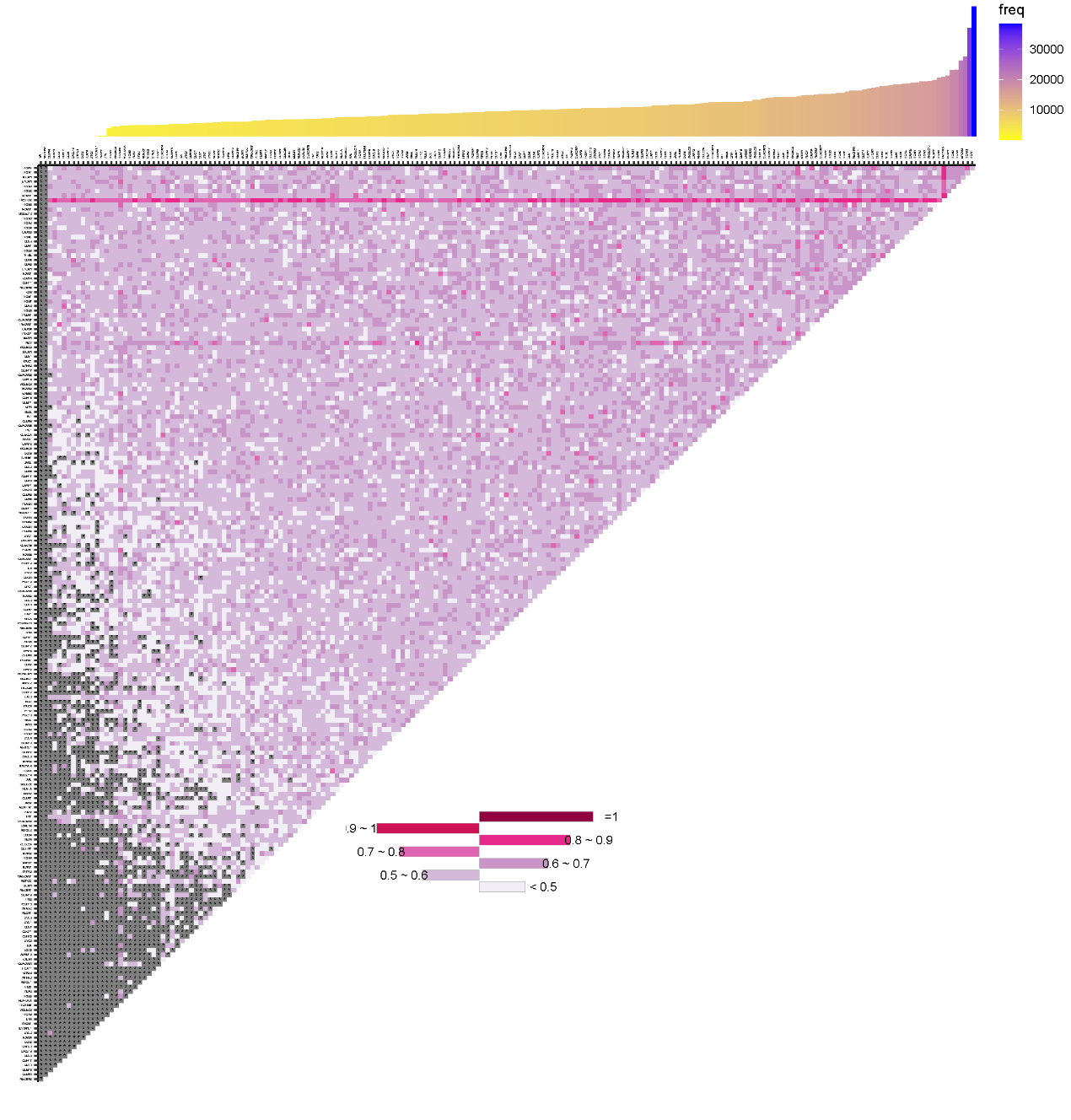


Figure S2. t-SNE plot for each sample in both groups. (Sample S17 was excluded.)


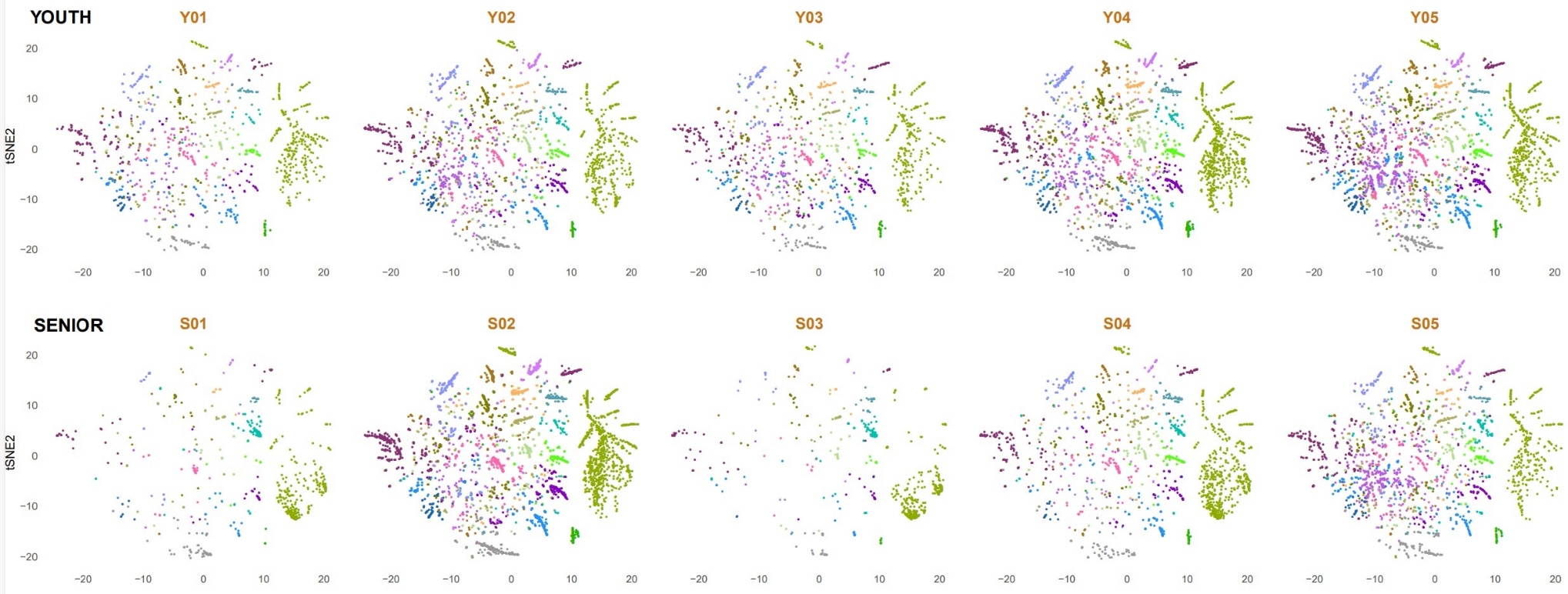


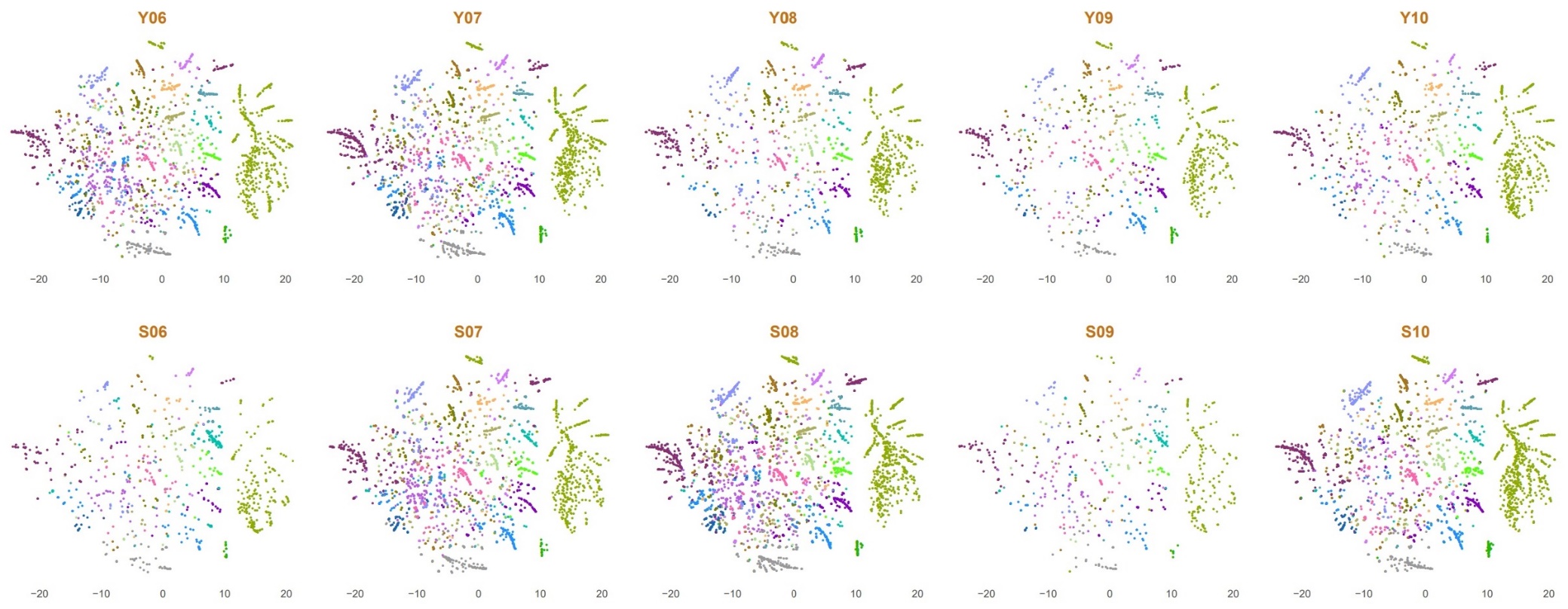


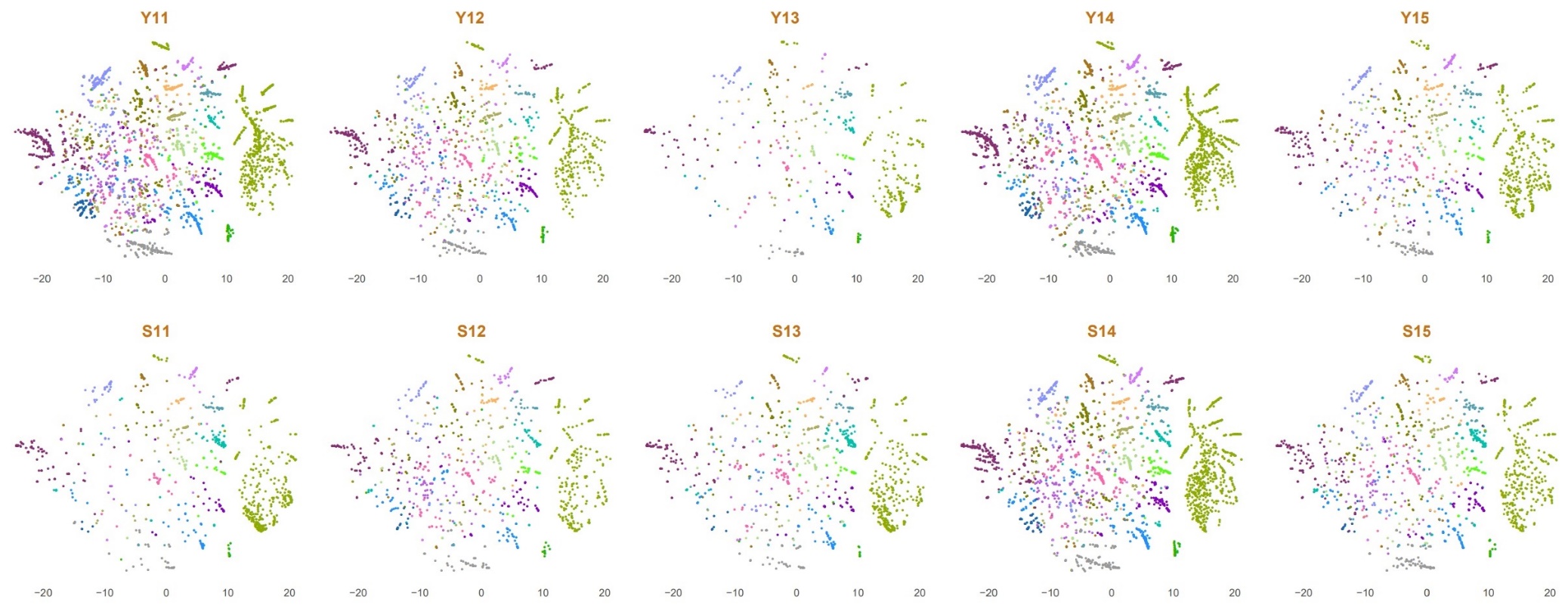


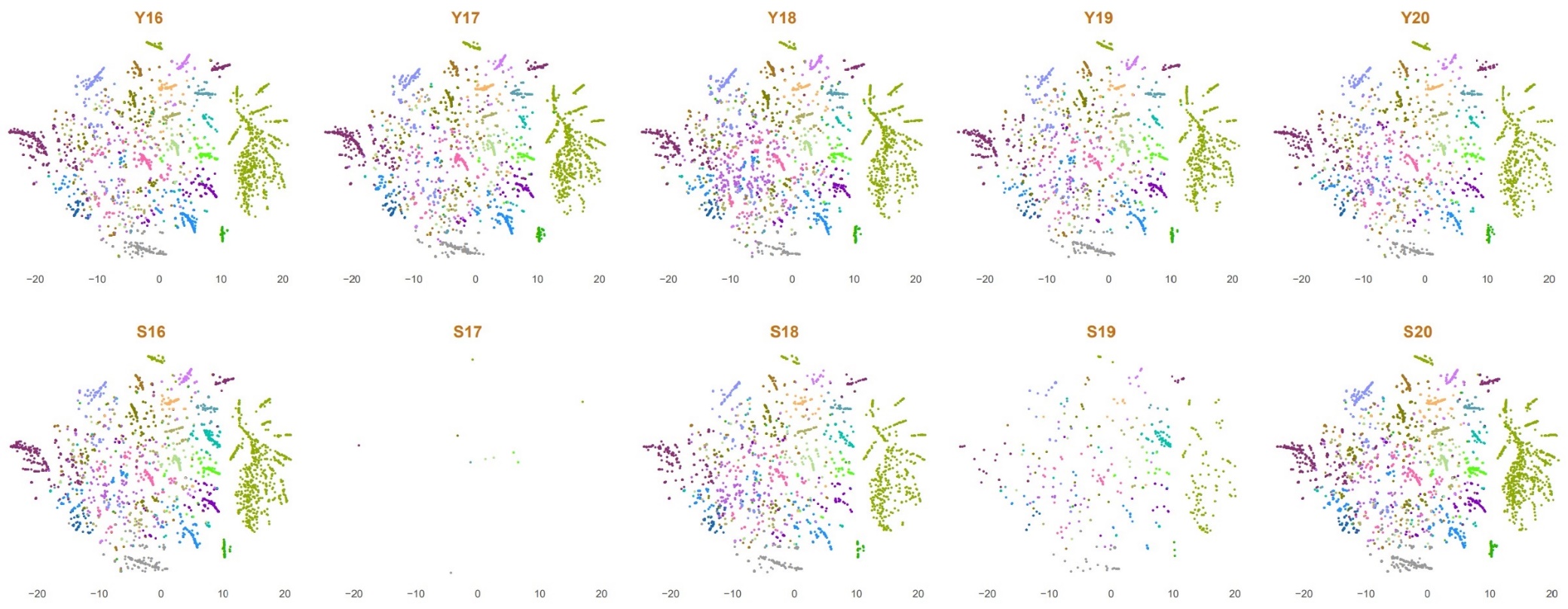


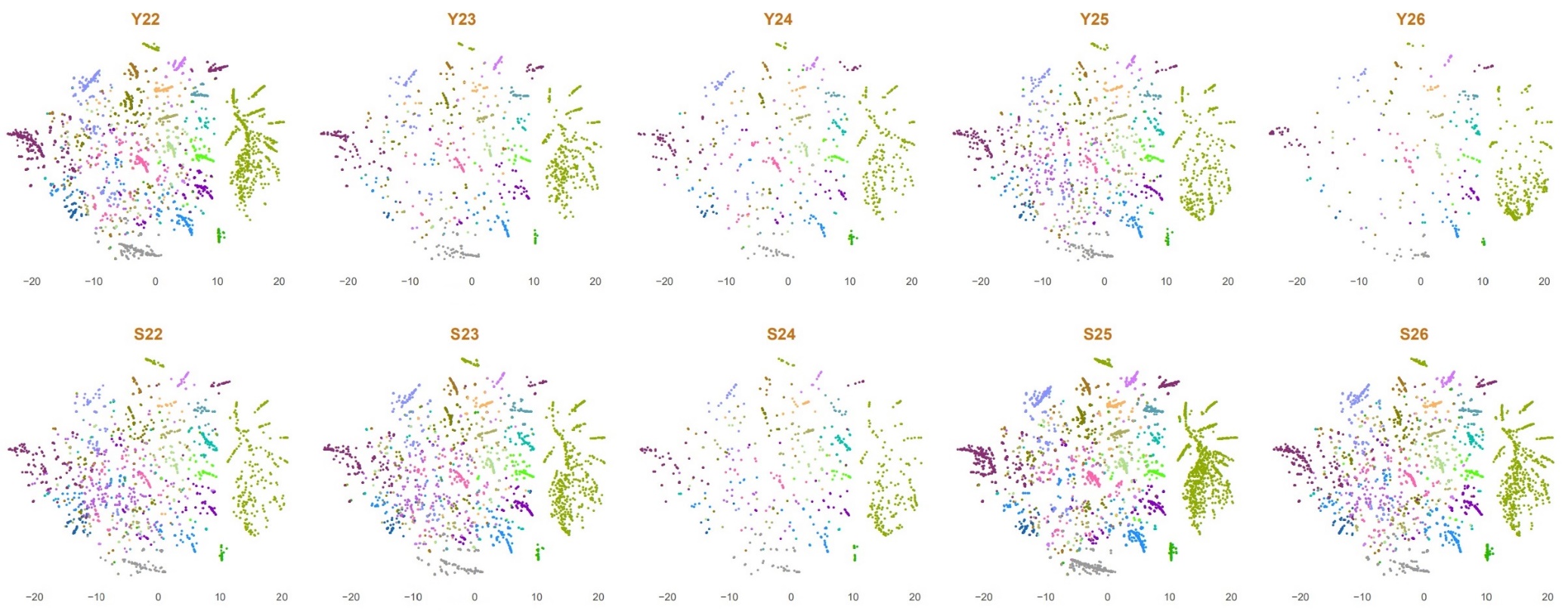


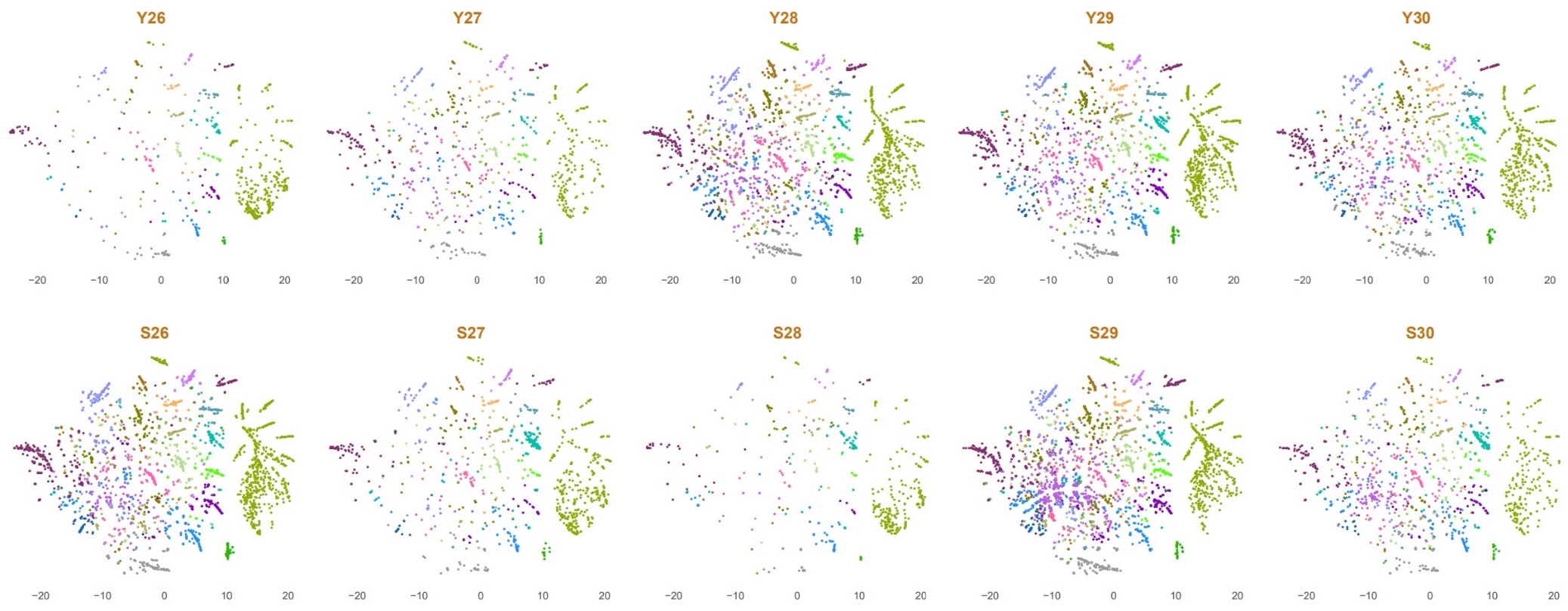


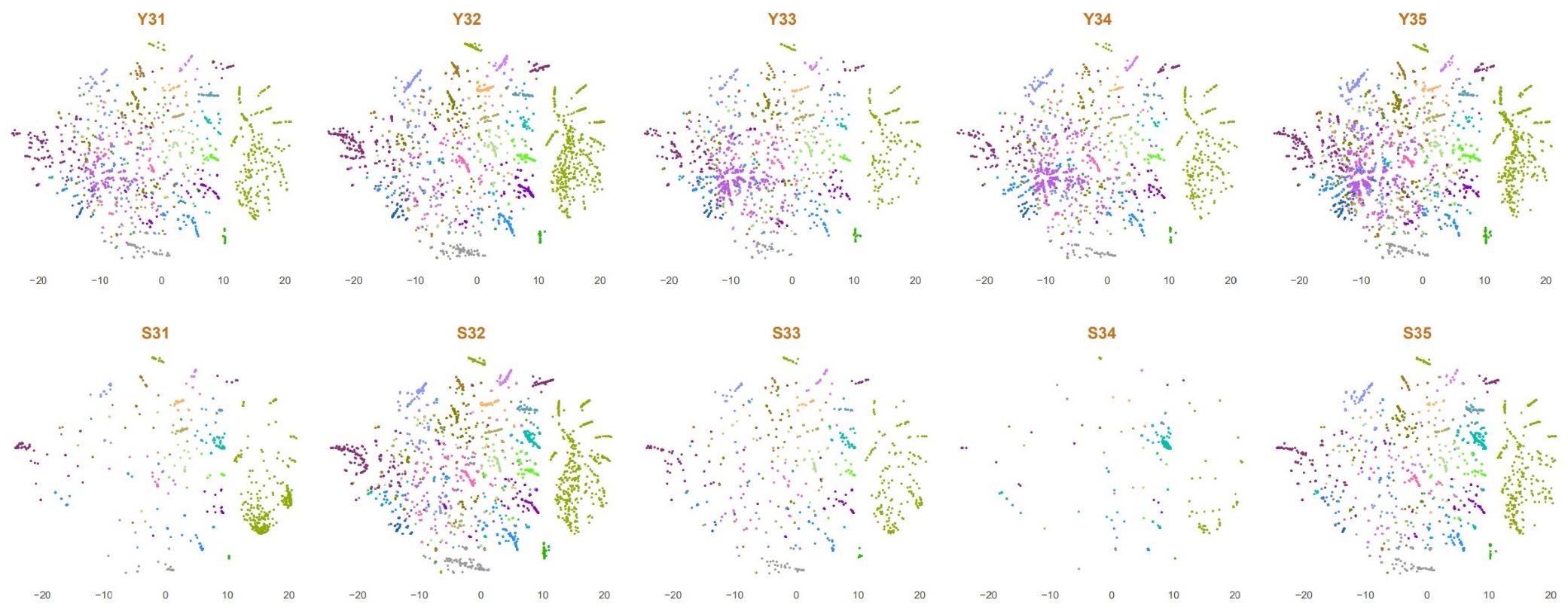


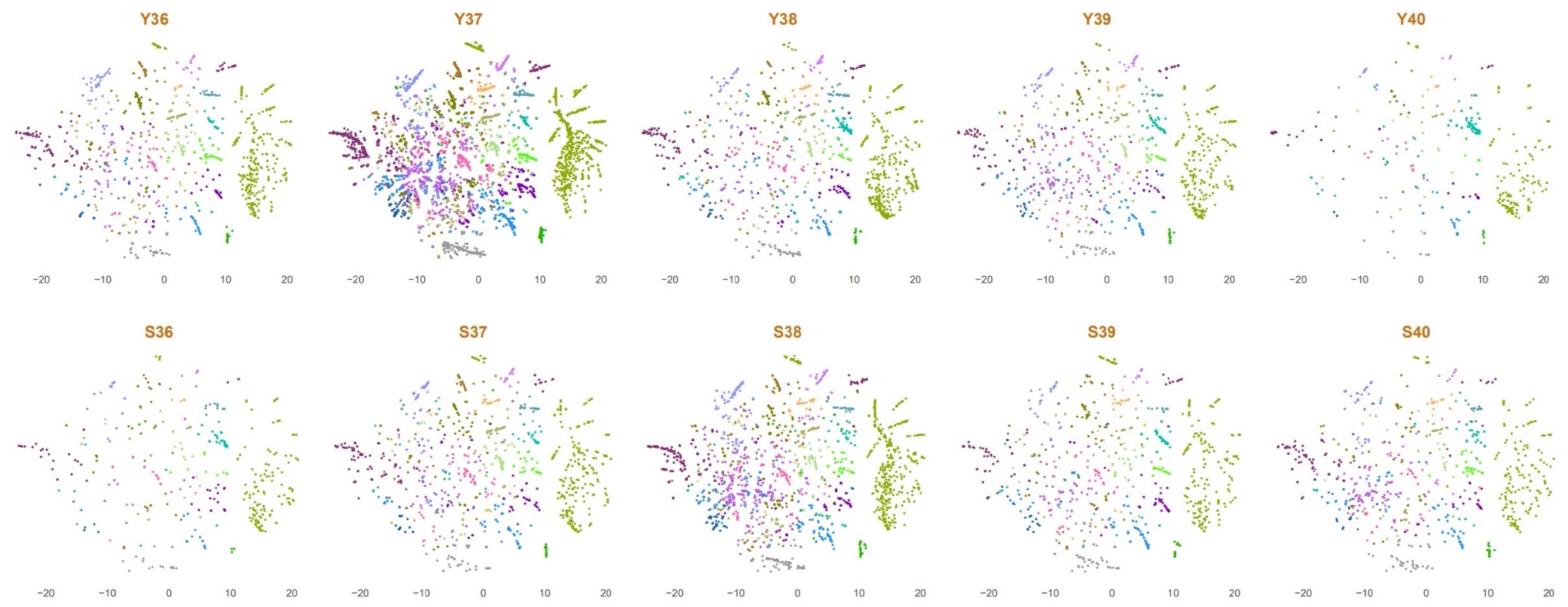


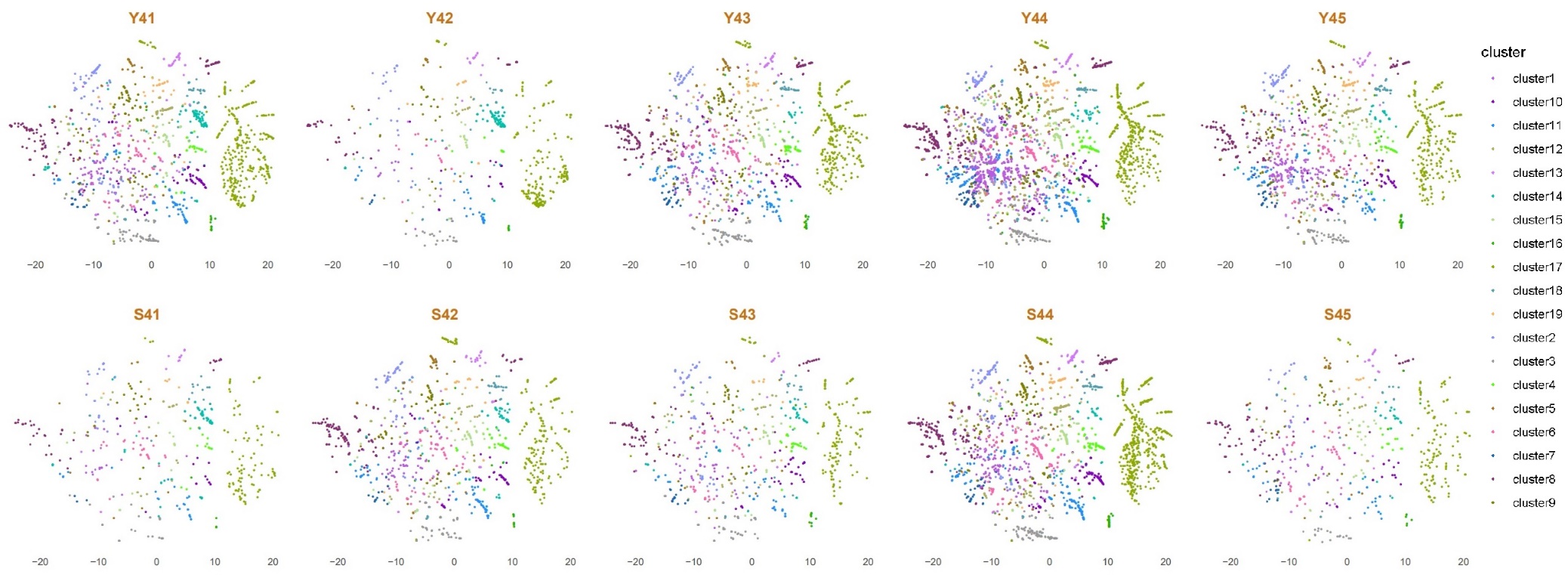


Figure S3. The expression of TACSTD2 mRNA in normal tissues and tumor tissues (UALCAN database).


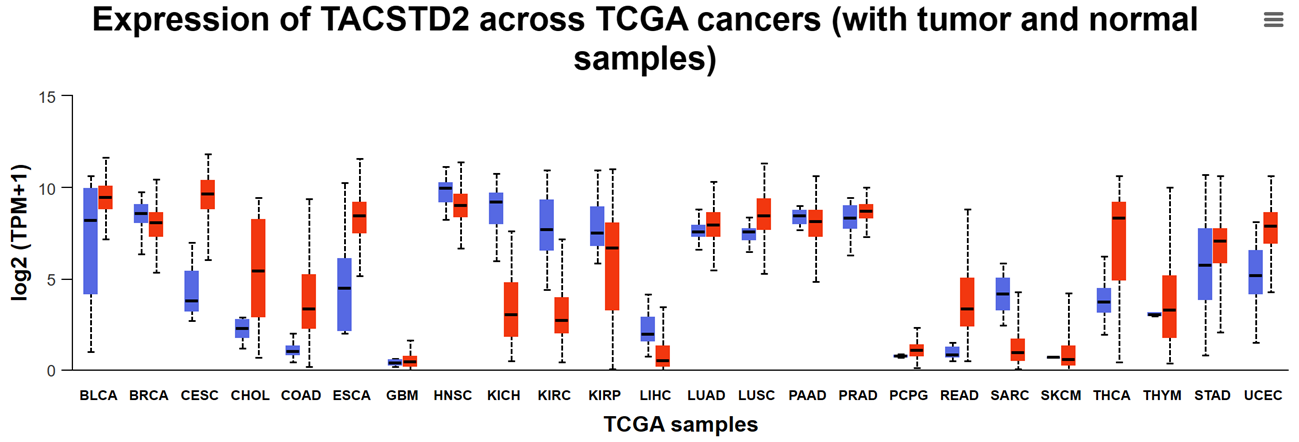

Supplement: Supplementary file 1 — Supplementary Material 1 [file 12951_2024_2456_MOESM1_ESM.docx]
